# Editorial: Sport as a tool for social inclusion, employment, and improved health

**DOI:** 10.3389/fspor.2023.1273858

**Published:** 2023-08-23

**Authors:** Chiara Corvino, Marcelo Moriconi, Cátia Miriam Costa, Chiara D’Angelo

**Affiliations:** ^1^ Università Cattolica del Sacro Cuore, Italy; ^2^ ISCTE, Instituto Universitario de Lisbona, Portugal

**Keywords:** sports, health, employment, social inclusion, sustainable development goals, employability

**Editorial on the Research Topic** Sport as a tool for social inclusion, employment, and improved health

## Intro: the topic

Currently, sports are well-known as a prominent means to address a wide spectrum of social goals. This acknowledgement of sports' social impact has been achieved through international acts and advocacy actions involving the United Nations (UN), the International Olympic Committee (IOC), sport federations, national governments and civic society over time (1). A recent significant step in formal recognition can be found in the 2030 Agenda for Sustainable Development through Resolution 70/1, which officially designates sports as an institutionalised sector contributing to sustainable development (2).

As a consequence of the identification of sports as catalysts for diverse social outcomes on the global and national agenda, the sport for development and peace (SDP) movement has emerged. SDP encompasses a wide range of actions, programs and studies wherein sports are utilised and investigated as means to promote non-sporting and peace building goals (3). SDP studies provide evidence regarding the variety of social benefits that can be achieved through sports, including: (i) health development (4–7); (ii) social inclusion (8–13); (iii) employment (14); (iv) sports integrity (15–17); (v) sports diplomacy (18–20); (vi) and sport, economy and crime (21).

Despite the richness of studies in the field, some open questions remain concerning the efficient use of sports as a tool for social and developmental purposes. First, the literature lacks frameworks and models guiding efficient sport-based program planning, structuring and management to maximize social outcomes (22–24). Secondly, the literature emphasizes the need to monitor and evaluate sport-based programs in order to explore “what works about sport” and “why it works” in specific contexts and conditions (22, 24–27). The collection of articles in this Research Topic contributes and enriches the debate on these open issues.

## The challenges

Most of the researches of the Research Topic draw upon data from diverse sport-based interventions aimed at various social goals (e.g., employment, youth development, economic, and cultural development) and implemented in different geographical areas, ranging from southern Africa to China, and from northern and southern Europe to Latin America. Despite the variety of social goals promoted by the programs analyzed in these studies and their geographical diversity, the articles provide similar evidence-based insights guiding: (i) the development of theoretically grounded sport initiatives; (ii) effective action planning; and (iii) efficient monitoring and evaluation of these initiatives.

Analyzing the results of a 6-month government-funded sport-for-development program implemented in Africa, with a focus on youth employability, Burnett demonstrates that engaging in such an initiative provided the participants with a high level of soft skills transferable to the working environment. The study advocates for a robust monitoring, evaluation, and learning (MEL) framework capable of capturing the nuances of the different contexts of program implementation.

In their contribution, Gozzoli et al. describe the participatory development of a training framework aimed at promoting sport coaches' awareness of their role in enhancing employability skills. The authors highlight the lack of clarity regarding the theoretical and methodological assumptions that guide the design of training courses and propose a constructivist approach to structure effective training programs for coaches.

Commers et al. analyze a program in Flanders aimed at transferring job skills to young people through sports. The research underscores the absence of clear definitions and theoretical operationalization of program outcomes, leading to gaps in efficiently monitoring and evaluating the participants’ progress within the intervention. The authors advocate for theory-based program planning, which could help outline the desired outcomes and impacts of the sport-based interventions more effectively.

Gadais et al. describe the experiences of Colombian youth from disadvantaged backgrounds and program managers who participated in an SDP intervention that took them from a local community sports club to the Olympic Games. The authors recommend the intensification of evaluation research on SDP programs, including close collaboration between academics, community-based organizations, donors, and the international community.

Finally, Kunlun et al. study the spatio-temporal distribution and evolution pattern of Chinese Go League Clubs and emphasize their cultural and economic impact at the local level.

## The results and the suggestions for future research on the topic

The comprehension of programme components, structure, planning and effective management remains a fundamental area for analysis to advance research and implementation in SDP (28). The articles compiled in this Research Topic propose two potential approaches for designing programs that effectively address contemporary social challenges. On one hand, Commers et al. advocate for grounding and planning sport-based programs based on the principles of the theory of change. Employing the theory of change offers the advantage of anticipating desired outcomes and impacts, providing a logical sequence of actions and inputs to achieve specific social changes. On the other hand, Gozzoli et al. suggest adopting less top-down approaches and promoting participatory methods for program development, involving local stakeholders and beneficiaries at all stages of the planning process. Future research should analyze and compare the opportunities and challenges of both approaches to advance the understanding of this field.

Effective program planning in sport-based initiatives is not only crucial for action delivery and impact but also has significant implications for monitoring and evaluation (M&E). There exists a circular relationship between planning, program implementation, and M&E. Burnett and Gadais et al. underscore the need to enhance M&E research in SDP. Future M&E investigations should encompass the nuanced impact of these interventions from various stakeholders' perspectives and should consider not only the individual effects of sport-based programs but also their broader economic and cultural implications (Kunlun et al.).
